# Oleogel-based drug delivery for the treatment of periodontitis: current strategies and future perspectives

**DOI:** 10.12688/f1000research.140173.1

**Published:** 2023-09-27

**Authors:** Shaswata Karmakar, Shashikiran Shanmugasundaram, Baishakhi Modak

**Affiliations:** 1 Department of Periodontology, Manipal College of Dental Sciences, Manipal Academy of Higher Education, Manipal, Karnataka, 576104, India; 2 Department of Oral and Maxillofacial Pathology and Oral Microbiology, Manipal College of Dental Sciences, Manipal Academy of Higher Education, Manipal, Karnataka, 576104, India

**Keywords:** Oleogels, Gels, Hydrogels, Local drug delivery, Periodontitis, Periodontics

## Abstract

Periodontitis is the chronic inflammation of tooth-supporting tissues that leads to loss of tooth support if untreated. Conventional therapy for periodontitis (mechanical removal of microbial biofilm and oral hygiene enforcement) is augmented by anti-microbial and anti-inflammatory drugs. These drugs are frequently delivered locally into the periodontal pocket for maximum efficiency and minimum adverse effects. The potential of oleogels for periodontal drug delivery has been discussed and further, the future scope of oleogel-based drug delivery systems in dentistry. An oleogel-based local drug delivery system offers several advantages over other systems. Superior mechanical properties (firmness and compressibility), muco-adhesion, shear thinning, thixotropy, controlled drug release and the ability to incorporate water-insoluble drugs clearly distinguish and highlight the potential of oleogels as periodontal local drug delivery systems. Bigels can combine the qualities of both hydrogels and oleogels to provide a more promising option for drug delivery. However, there is limited evidence concerning oleogels as local drug delivery agents in periodontics. Further studies are needed to discern the clinical efficacy of oleogel-based drug delivery systems.

## Introduction: Drug delivery in periodontics

**Figure 1.**
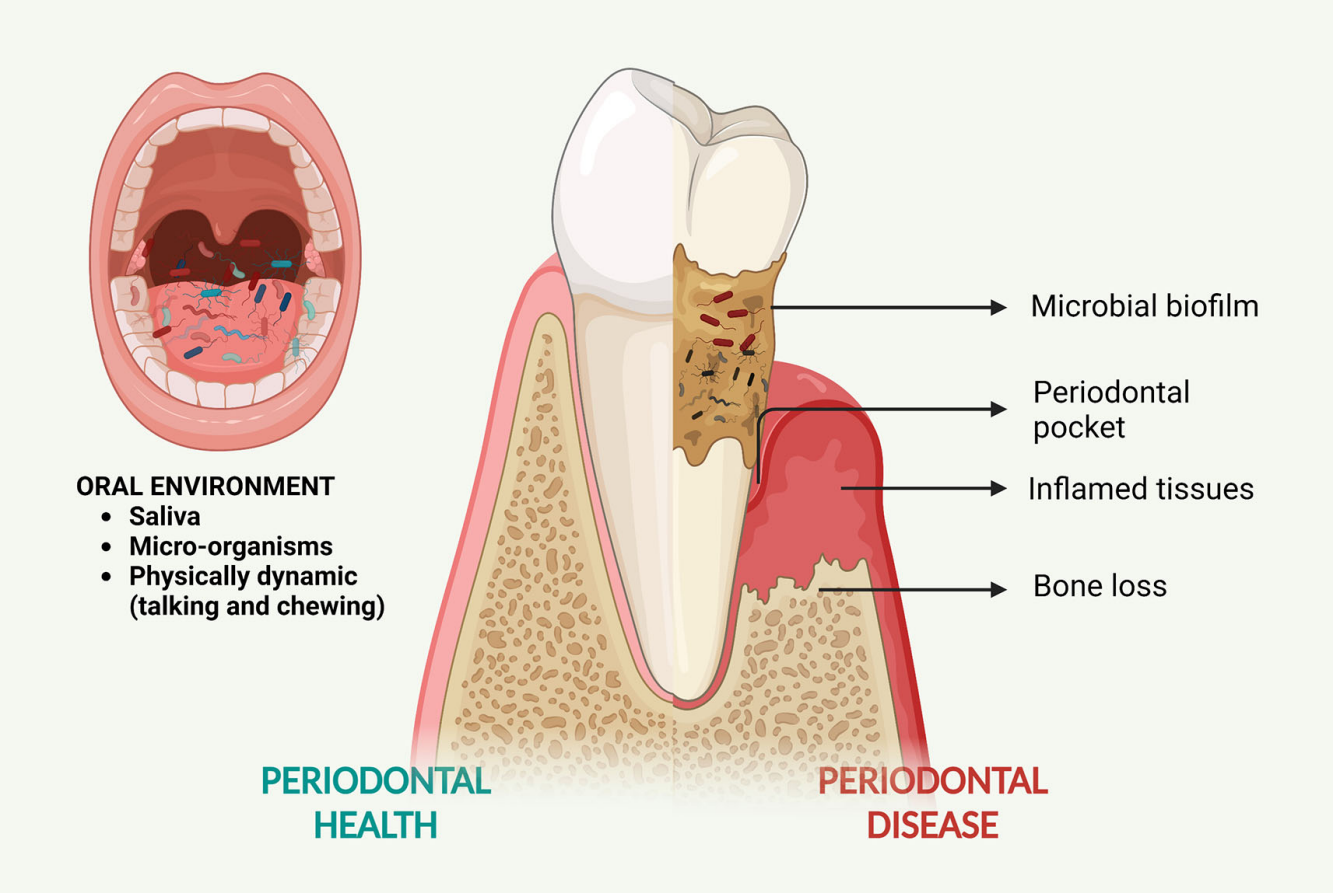
Periodontal disease and the oral environment. The microbial biofilm that accumulates on the teeth causes chronic inflammation of tissues around the tooth leading to the formation of periodontal pockets and a gradual loss of tooth support. The oral cavity encounters a constant flow of saliva and frequent physical movements due to talking and chewing. The oral cavity also hosts a diverse microbiome. Created with BioRender.com.

Periodontics is the branch of dentistry that deals with the prevention, diagnosis and treatment of diseases affecting the tissues that surround and support the teeth. The World Health Organization (WHO) estimates that periodontal diseases affect around 19% of adults around the world. ^1^ Periodontitis is the chronic inflammation of the alveolar bone and other soft tissues surrounding the teeth that leads to gradual loss of tooth support, ultimately leading to tooth loss, if untreated. ^2^ This chronic inflammation is triggered by dysbiotic microbial biofilm accumulating around the teeth. Periodontitis often results in periodontal pockets (deepening of the gingival sulcus) around the teeth ( Figure 1). These areas act as biofilm retentive niches that are inaccessible to hygiene procedures (brushing and flossing) where biofilm continues to accumulate leading to a slow but continuous destruction of tissues. ^3^ The host immuno-inflammatory mediators such as cytokines and prostaglandins also play a significant role in the disease progression and tissue damage. ^4^

Periodontal therapy aims to remove the accumulated biofilm (mechanical debridement), disinfect the pocket region, resolve inflammation, and enforce optimal hygiene habits in the patients. The main goal of periodontal therapy is to eliminate or control the microbial biofilm and restore the health and function of the periodontium ( Figure 1). Various antimicrobial and anti-inflammatory drugs have been used along with mechanical debridement (professional removal of microbial biofilm) for the treatment of periodontitis; and delivering these drugs locally into the pocket region provides the best efficiency in terms of drug concentration at the site while eliminating systemic side effects in patients. Oral administration of drugs has some inherent drawbacks such as reduced bioavailability of the drug at the required site (due to first-pass metabolism in the liver) and systemic side effects. To overcome these drawbacks, clinicians often choose to locally deliver the drugs into the inflamed sites. ^5^

**Figure 2.**
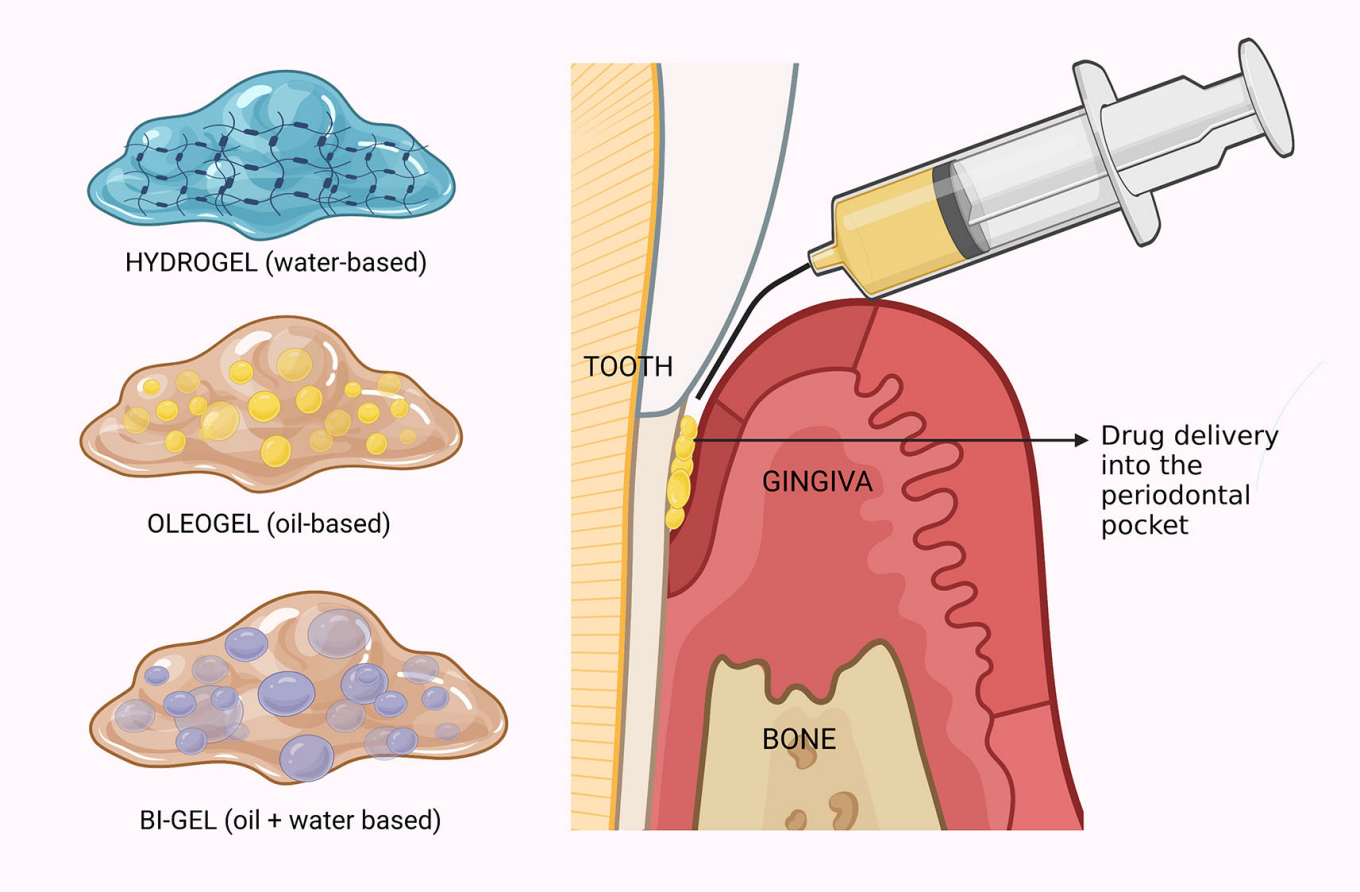
Gel-based local drug delivery for periodontal therapy. The periodontal pocket is a deep narrow space around the tooth affected by periodontitis. The pocket is bounded on one side by the tooth and the other side by the gingival soft tissue. Therapeutic agents (anti-microbial or anti-inflammatory) loaded in either of the three gel systems are delivered locally into the periodontal pocket to control the inflammation. Created with BioRender.com.

The periodontal pocket is a narrow crevice encircling the tooth. The region is bounded by soft tissue on one side and hard tissue (tooth) on the other. ^6^ The therapeutic agents delivered into the periodontal pocket must be retained in the region for a long time without getting displaced by the continuous flow of saliva and the constant physical movements of the oral cavity. Hence, the delivery system needs to be adhesive to the pocket wall ( Figure 2). Also, the release of the drug into the region should be slow and sustained to resolve inflammation. ^6^ One of the major challenges in periodontics is to efficiently deliver therapeutic agents to the inflamed sites around the teeth and maintain their concentration for sufficient time. The oral cavity is a physically dynamic environment with a constant flow of saliva, which makes local delivery of drugs into the periodontal environment a challenge ( Figure 1). Therefore, there is a need for developing novel drug delivery systems that can provide sustained and controlled release of drugs in this dynamic environment. ^5^

Among the various types of local drug delivery systems, gels have received considerable attention for periodontal applications. ^7^ Gels are semi-solid systems that consist of a liquid phase entrapped within a three-dimensional network of cross-linked polymers or solid particles. ^7^^,^^8^ Gels are biocompatible, can be administered easily, and can adapt to different shapes in the periodontal region. ^7^ Depending on the nature of the liquid phase, gels can be classified into hydrogels, oleogels and bigels ( Figure 2). Hydrogels are gels that contain water as the liquid phase. They are widely used for drug delivery purposes, as they can swell and release drugs in response to various stimuli, such as pH, temperature, enzymes, or electric fields. However, hydrogels also have some drawbacks, such as rapid drug release, low mechanical strength, poor stability, and inability to incorporate hydrophobic drugs. ^8^ Oleogels have oil as their liquid phase and have been explored for food, cosmetics, and pharmaceutical applications. Oleogels have shown potential for drug delivery due to their unique properties, such as high solubility and stability of lipophilic drugs, low water activity and microbial growth, tunable rheology, and texture, and injectability with a needle. ^9^ Bigels are gels that contain both water and oil as the liquid phases. They are relatively new gels that combine the benefits of hydrogels and oleogels while overcoming their drawbacks.

In this review, we explore the current state of knowledge on oleogels for drug delivery in periodontics; provide an overview of the recent advances in oleogels as local drug delivery systems for periodontitis; and discuss the challenges and future perspectives of oleogel-based drug delivery systems.

## Oleogels in periodontics

Gel formulations, particularly hydrogels, have drawn a lot of attention among these local delivery systems for the treatment of periodontitis. Non-steroidal anti-inflammatory drugs (NSAIDs) and antibacterial agents are frequently delivered *via* hydrogels made of sodium carboxymethyl cellulose, chitosan, polyethene oxide, xanthan gum, and polyacrylic acid to treat periodontitis. The ability of hydrogels to adhere to the mucosa in the periodontal pocket and then be quickly removed by typical catabolic routes demonstrates their excellent biocompatibility and mucoadhesive qualities. ^10^^,^^11^ However, the major drawback of using hydrogels is that they are less compatible with lipophilic drugs.

Oleogels are formed by adding a structuring agent to an edible oil to create a three-dimensional network that entraps the oil molecules. The structuring agent can be either a low-molecular-weight gelator, such as fatty acids or waxes, or a high-molecular-weight gelator, such as polymers or proteins. Bigels can be synthesized by mixing organogel (oil phase) and hydrogel (aqueous phase) components in different proportions and methods. Bigels can provide improved drug loading capacity, release kinetics, rheological properties and biocompatibility compared to hydrogels or oleogels alone. ^12^ Oleogels can enhance the penetration of drugs due to their lipophilic nature. They have been used to deliver water-insoluble drugs like tetracycline, metronidazole, metronidazole benzoate, and their combinations. ^13^ Bigels, another system of drug delivery, is a biphasic system created by combining water-based hydrogels and oil-based oleogels. So, it is possible to incorporate and deliver both lipophilic and hydrophilic drugs at the same time.

In the past, oleogels have been used for periodontal drug delivery. One study evaluated “Elyzol ^®^ 25%” dental gel (Colgate-Palmolive (UK) Limited), which contains sesame oil, glyceryl monooleate, and metronidazole benzoate. ^14^ When the pocket was filled with it, it flowed smoothly *via* the applicator to the location. This offers a simple and efficient method of administration. ^14^ “ATRIDOX ^®^ gel” (Atrix Lab, USA), another local drug delivery agent is composed of poly- dl-Lactide dissolved in a biocompatible solvent *N*-methyl-2-pyrrolidone loaded with 10% doxycycline hyclate. Sol-gel transition and the formation of a semisolid structure on encountering Gingival Crevicular Fluid (GCF) is a characteristic of this system that is based on water-free mixtures of lipids such as glycerol monooleate and sesame oil. Studies were conducted using this gel for the treatment of periodontitis and the results showed that 250 mg/ml of drug was present in GCF for seven days indicating sustained drug delivery. ^15^^,^^16^ A study developed and evaluated a metronidazole gel and reported adequate muco-adhesiveness and controlled drug release. ^17^ Another recent study evaluated a thermoreversible metronidazole gel and reported sustained antibacterial action. ^18^ The delivery of a combination of amoxicillin and metronidazole using a lipid-based gel system showed sustained anti-bacterial efficacy ^19^ ( Table 1).

**Table 1.** Studies evaluating oleogels as a drug delivery method for periodontitis. SRP (Scaling and Root Planing); GCF (Gingival Crevicular Fluid).

Sl no	Author and year of study	Type of study	Purpose of study	Results of study
1	Sallam A *et al.* (2015) ^17^	*In vitro*	Preparation of controlled release mucoadhesive metronidazole oleogel	The prepared gel was mucoadhesive, injectable and showed sustained-release properties.
2	Yadav E *et al.* (2022) ^18^	*In vitro*	Preparation of thermoreversible metronidazole oleogel	Excellent long-term antimicrobial activity was observed against potent periodontopathogens
3	Griffiths G *et al.* (2000) ^14^	Clinical trial	Determination of efficacy of 25% metronidazole oleogel and comparison of it with SRP	Significant improvement of the clinical parameters for periodontal treatment
4	Javali M *et al.* (2012) ^15^	Clinical trial	Determination of efficacy of 10% doxycycline hyclate gel	A significant concentration of the drug was found in GCF even after seven days of placement
5	Léber A *et al.* (2019) ^19^	*In vitro*	Preparation and evaluation of mucoadhesive, monophase lipid-based gel for delivery of amoxicillin, metronidazole and Zinc hyaluronate for periodontitis treatment	Sustained local delivery of the prepared lipid system showed higher efficacy against periodontopathogens for amoxicillin than metronidazole and Zinc hyaluronate
6	Beg S *et al.* (2020) ^35^	Clinical trial	Development of a novel nanoparticle-containing gel formulation containing moxifloxacin for local delivery in periodontitis patients	Reduction in probing pocket depth and decrease in IL-1 and TNF-α levels were observed
7	Toskic-Radojicic M *et al.* (2005) ^36^	*In vitro*	Comparison of different antimicrobial-containing oleogels for the treatment of periodontitis	Significant antimicrobial activity was observed for oleogels prepared with 2.5% tetracycline, 25% metronidazole and a combination of these two drugs.

There are a limited number of studies comparing different types of gel systems that can be used for local drug delivery for periodontitis. Hamed *et al.*, conducted a study investigating the local delivery of NSAIDs to treat periodontitis using different gel formulations. ^12^ Gel formulations were characterized in terms of their various properties (flow behavior, viscoelasticity and bio-adhesive properties, *in vitro* drug release). The results of the study showed that rheological and bio-adhesive properties were greatly influenced by the type of gel formulations. Oleogels and bigels showed superior properties such as viscoelasticity, bio-adhesion and controlled-drug release when compared to hydrogels. The study concluded that bigels and oleogels showed the greatest promise as a potential local drug delivery system for treating periodontitis. ^12^ Promising results have also been obtained with several oleogels for the delivery of 2.5% tetracycline, 25% metronidazole, 40% metronidazole benzoate, as well as a combination of tetracycline and metronidazole benzoate. ^20^

Another study compared the efficacy of hydrogels, oleogels, and bigels loaded with metronidazole for treating periodontitis. ^21^ Bigels and Oleogels showed superior properties like shear-thinning (a property that enables the gel to form thin layers and to be injected by syringes), thixotropy (the gel is rendered more viscous under stress and less viscous in the absence of stress), bio-adhesiveness (the ability of the gel to adhere to the mucosa). Oleogels showed better mechanical properties like firmness, compressibility, and adhesiveness when compared to bigels and hydrogels. However, among the three gel systems, oleogels showed the least drug release and bigels showed the highest drug release. The study concluded that bigels and oleogels are promising local drug delivery systems to treat periodontitis. ^21^ A 2022 study formulated and evaluated olive oil and mustard oil-based oleogels as drug-delivery agents loaded with metronidazole. The prepared oleogels showed thermoreversible behavior, shear-thinning, and pseudo-plasticity. The gels also showed pH-responsive controlled drug release. The study concluded that these gels could serve as excellent tools for drug delivery. ^22^ Overall, the superior properties of oleogels and bigels can potentially enable a more efficient and robust drug delivery to the periodontal region and aid in successful therapy.

## Other applications of oleogels in dentistry

Oil-based or lipid-based formulations can be used to treat many other mucosal and inflammatory dental diseases and conditions and have been employed more frequently during the past few decades for their applicability in a variety of treatment aspects such as gene delivery, cancer therapy, and infectious disorders. ^23^ They are preferred drug carriers due to their capacity to enclose, transport and safeguard molecules with a variety of physical and chemical properties. ^23^ Liposomes, being the active component of lipid-based compounds, are tiny, adaptable lipid-based vesicles that can enclose hydrophilic, lipophilic, and amphiphilic substances. Increased biocompatibility and site-specificity, increased therapeutic index and less toxicity are some of its important properties, making it suitable for drug administration in dentistry. ^24^ In recent years, a great number of lipid-based formulations have been developed for oral drug delivery to treat various diseases, including diseases of oral-dental origin. ^25^

Several diseases of the oral mucosa like erythema multiforme, pemphigus vulgaris, lichen planus, and bullous pemphigoid of the oral mucosa need topical steroid delivery. ^26^^–^^28^ Oleogels can provide a superior alternative to currently existing steroid-delivery systems. Local anesthetic gels containing Lidocaine or Benzocaine are frequently used for the symptomatic treatment of oral ulcers. ^29^ An oleogel-based delivery system, with improved bio-adhesiveness and controlled release, could provide sustained action at ulcerated sites.

Oral cancer is one of the most prevalent health concerns around the globe. Chemotherapeutic drugs frequently have unfavorable side effects on noncancerous tissues when used to treat oral cancer. Drug resistance and metastasis result from cancer therapies’ being unable to completely eradicate malignant cells. ^30^ However, lipid-based drugs can dispense an appropriate amount of drug to the tumor cells, making treatment more effective. ^30^ Doxil ^®^ was the first FDA (Food and Drug Administration, USA)-approved liposomal doxorubicin for the treatment of oral cancer. Additionally, lipid-based drugs may enhance the activity of certain drugs, bypass drug-resistance mechanisms, and reduce the toxicity associated with them, especially for drugs like antibiotics. ^30^ A wide range of antibiotics are used in dentistry. Rukavina *et al.*, developed azithromycin-loaded liposomes that improved the antibiotic’s activity against various oral infections caused by *Staphylococcus aureus.* The formulation also retained the drug more efficiently and prevented oral biofilm formation. ^31^ In a recent study, Ribeiro *et al.*, showed their hybrid liposomal formulation’s improved *in vitro* antimicrobial activity against various drug-resistant bacterial strains. ^32^

## Current challenges and future perspectives

Oleogel-based drug delivery systems seem to offer numerous advantages such as delivery of water-insoluble drugs, enhanced muco-adhesiveness, sustained delivery, shear thinning, and thixotropy; oleogels have the potential to serve as a superior alternative to hydrogel-based drug delivery. However, the formulation of oleogels needs to be standardized and optimized for oral drug delivery and should be tailored for specific drugs. The choice of the appropriate oil phase and gelator can enhance the qualities of the oleogel. The safety of oleogels needs to be studied further; biodegradability, biocompatibility, and potential for toxicity and adverse reactions need to be elucidated before clinical use. Future clinical trials need to study the efficacy of oleogel-based drug delivery systems in humans.

Bigels can be a significant improvement over the characteristics of oleogels as they can combine the benefits of both hydrogels and oleogels. But the ideal ratios and proportions of aqueous and oil phases need to be studied in the future as they can significantly influence the behavior and characteristics of these gel systems. ^12^ Temperature-sensitive and pH-sensitive smart oleogel systems (like their hydrogel counterparts) can greatly enhance the treatment of diseases like periodontitis as the periodontal pocket region has variable temperature and pH in accordance with health and disease states. ^33^ Nanogels can significantly increase the drug loading capacity with increased permeability. ^34^ The development of stimuli-responsive smart oleogel systems and oil-based nano-gels (nano-oleogels) in the future can augment the potential of oleogels for drug delivery.

## Conclusions

Preliminary evidence suggests that oleogels and bigels offer several advantages over hydrogels. The ability to incorporate water-insoluble drugs, higher muco-adhesion, controlled drug release, and superior mechanical properties highlights the potential of oleogels to be used as an alternative drug delivery system in periodontics. Oleogels and bigels also show shear-thinning and thixotropic behavior that can aid in their placement and retention in the periodontal pocket. However, several aspects of the formulation such as the ideal choice of the oil phase, gelators and other additives, especially for oral mucosal drug delivery need to be studied. The biodegradability of oleogels and the possibility of toxicity also need further research. Currently, there is limited evidence concerning oleogels and their efficacy in periodontal drug delivery. Future research needs to focus on the knowledge gaps in the literature and should address the safety and efficacy of oleogel-based periodontal drug delivery in humans.

## Data Availability

No data are associated with this article.
