# Supplementary material for: Oleogel-based drug delivery for the treatment of periodontitis: current strategies and future perspectives
Source: F1000Res. 2023 Sep 27;12:1228. [Version 1] doi: 10.12688/f1000research.140173.1 (PMC11058454; doi:10.12688/f1000research.140173.1)
Supplement: Peer review history

# Reviewer report: Reviewer response for version 1

**DOI:** 10.5256/f1000research.153504.r267725

**Published:** 2024-05-06

**Authors:** Dana Gabriela Budală, Ionut luchian

**Affiliations:** 1 “Grigore T. Popa” University of Medicine and Pharmacy, Iași, Romania; 2 periodontology, umf gr t.popa, IASI, Iasi, Romania

Oleo gels are emerging as a promising option for the localized treatment of periodontitis. A thorough examination of oleo gels as local drug delivery agents has been conducted, highlighting their uses, advantages, and limitations. The article provides a deeper understanding of the role of oleo gels in dental and periodontal care. Oleo gels show significant potential for future use in the targeted treatment of periodontitis.

Is the review written in accessible language?

Yes

Are all factual statements correct and adequately supported by citations?

Yes

Are the conclusions drawn appropriate in the context of the current research literature?

Yes

Is the topic of the review discussed comprehensively in the context of the current literature?

Yes

Reviewer Expertise:

prosthodontics

We confirm that we have read this submission and believe that we have an appropriate level of expertise to confirm that it is of an acceptable scientific standard.

## References

[1] : Are Local Drug Delivery Systems a Challenge in Clinical Periodontology?. *J Clin Med* .2023;12(12) : 10.3390/jcm12124137 10.3390/jcm12124137 37373830 PMC10298898

# Reviewer report: Reviewer response for version 1

**DOI:** 10.5256/f1000research.153504.r253604

**Published:** 2024-04-29

**Authors:** Shikha Sharma

**Affiliations:** 1 SGT DENTAL COLLEGE, GURUGRAM, India

A comprehensive review regarding the ole gel as local drug delivery agent has been discussed. the indications, advantages  and disadvantages have been elaborated.  The article provides an insight into the dental and periodontal indications of the oleo  gels.

 oleo gels forms a promising future for the treatment of periodontitis as a local drug delivery agent.

 The article clearly draws the importance of the lipophilic nature of the oleogels and their importance in delivering some of the drugs used in the treatment of periodontitis like metronidazole etc. The Article comprehensively describes the use of oleogels in dentistry including in periodontics .The article briefs about the current trends, challenges  and future perspective of olegels  in periodontics

 Major

 The article doesn't discuss the comparison between the mechanism, advantages, indications of different mode of the delivering of LDD apart from olegel ,just name has been mentioned i.e. Hydrogel and bigels , further elaboration about the same can be done , to bring about the importance and point of interest in regards with the oleogels proving it be more beneficial than the counter parts.

 The article has in depth beautifully  explained about the oleogels , describing its features , structures , researches done in periodontics and general dentistry 

 minor 

 article in depth has described the used of oleogels from past till present .

Is the review written in accessible language?

Yes

Are all factual statements correct and adequately supported by citations?

Yes

Are the conclusions drawn appropriate in the context of the current research literature?

Yes

Is the topic of the review discussed comprehensively in the context of the current literature?

Yes

Reviewer Expertise:

periodontics

I confirm that I have read this submission and believe that I have an appropriate level of expertise to confirm that it is of an acceptable scientific standard.

## References

[1] : Guar gum and sesame oil based novel bigels for controlled drug delivery. *Colloids Surf B Biointerfaces* .2014;123: 10.1016/j.colsurfb.2014.09.056 582-92 10.1016/j.colsurfb.2014.09.056 25444661

[2] : Formulation development of novel in situ nanoemulgel (NEG) of ketoprofen for the treatment of periodontitis. *Drug Deliv* .2016;23(1) : 10.3109/10717544.2014.907842 154-66 10.3109/10717544.2014.907842 24786482

[3] : Development of satranidazole mucoadhesive gel for the treatment of periodontitis. *AAPS PharmSciTech* .2009;10(3) : 10.1208/s12249-009-9260-z 716-23 10.1208/s12249-009-9260-z 19479385 PMC2802163

# Reviewer report: Reviewer response for version 1

**DOI:** 10.5256/f1000research.153504.r216725

**Published:** 2023-10-19

**Authors:** Dhirendra Kumar Singh

**Affiliations:** 1 Department of Periodontology, Kalinga Institute of Dental Sciences, KIIT Deemed to be University, Bhubaneswar, Odisha, India

The manuscript is well formulated, and structured with comprehensive information on the topic, as the non-invasive and non-surgical periodontal treatments are the need of the hour. The present manuscript will definitely help future researchers in gathering the cumulative information on local drug delivery systems from just one article.

Is the review written in accessible language?

Yes

Are all factual statements correct and adequately supported by citations?

Yes

Are the conclusions drawn appropriate in the context of the current research literature?

Yes

Is the topic of the review discussed comprehensively in the context of the current literature?

Yes

Reviewer Expertise:

Periodontology, Periodontal microbiology, Oral Implantology,

I confirm that I have read this submission and believe that I have an appropriate level of expertise to confirm that it is of an acceptable scientific standard.

## References

[1] : Local drug delivery in periodontitis treatment: A review of contemporary literature. *Dent Med Probl* .2018;55(3) : 10.17219/dmp/94890 333-342 10.17219/dmp/94890 30328312

[2] : Local drug delivery systems in the management of periodontitis: A scientific review. *J Control Release* .2019;307: 10.1016/j.jconrel.2019.06.038 393-409 10.1016/j.jconrel.2019.06.038 31255689

[3] : Injectable hydrogels with high drug loading through B-N coordination and ROS-triggered drug release for efficient treatment of chronic periodontitis in diabetic rats. *Biomaterials* .2022;282: 10.1016/j.biomaterials.2022.121387 121387 10.1016/j.biomaterials.2022.121387 35093823

[4] : Local Drug Delivery Systems in the Treatment of Periodontitis: A Literature Review. *J Int Acad Periodontol* .2015;17(3) :82-90 26373225

[5] : Evaluation of Different Local Drug Delivery Systems in the Management of Chronic Periodontitis: A Comparative Study. *J Contemp Dent Pract* .2020;21(3) :280-284 32434975
